# Therapeutic Potential of Antibody-Drug Conjugate-Based Therapy in Head and Neck Cancer: A Systematic Review

**DOI:** 10.3390/cancers13133126

**Published:** 2021-06-22

**Authors:** Vittoria Perrotti, Vito Carlo Alberto Caponio, Marco Mascitti, Lorenzo Lo Muzio, Adriano Piattelli, Corrado Rubini, Emily Capone, Gianluca Sala

**Affiliations:** 1 Department of Medical, Oral and Biotechnological Sciences, Gabriele d’Annunzio University of Chieti-Pescara, 66100 Chieti, Italy; adriano.piattelli@unich.it; 2 Department of Clinical and Experimental Medicine, University of Foggia, 71100 Foggia, Italy; vitocarlo.caponio@unifg.it (V.C.A.C.); lorenzo.lomuzio@unifg.it (L.L.M.); 3 Department of Clinical Specialistic and Dental Sciences, Marche Polytechnic University, 60121 Ancona, Italy; marcomascitti86@hotmail.it; 4 Fondazione Villa Serena per la Ricerca, Città S. Angelo, 65121 Pescara, Italy; 5 Casa di Cura Villa Serena, Città S. Angelo, 65121 Pescara, Italy; 6 Department of Biomedical Sciences and Public Health, Marche Polytechnic University, 60121 Ancona, Italy; c.rubini@univpm.it; 7 Department of Innovative Technologies in Medicine & Dentistry, University of Chieti-Pescara, 66100 Chieti, Italy; emily.capone@unich.it (E.C.); g.sala@unich.it (G.S.); 8 Center for Advanced Studies and Technology (CAST), Via Polacchi 11, 66100 Chieti, Italy

**Keywords:** antibody-drug conjugate, clinical trials, head and neck cancer, linkers, payload

## Abstract

**Simple Summary:**

Head and neck cancer (HNC) is a complex and extremely heterogeneous disease that includes a wide variety of cancer subtypes. Despite notable advances in understanding the molecular mechanisms involved in the disease, which allowed the increase of the therapeutic armamentarium, HNC treatment remains very challenging. In fact, to date the average 5-year survival rate for this disease is around 65%; hence, HNC continues to be one of the most aggressive solid tumors. Surgical removal is the first treatment of choice for HCN; however, in addition to this treatment modality, a broad spectrum of new therapies has been developed so far, ranging from multimodal chemotherapy to targeted and immune-therapy, mainly through the use of antibodies. In this work, we systematically reviewed the progress obtained in antibody-drug conjugate (ADC) development for the treatment of HNC.

**Abstract:**

Background: Antibody-drug conjugates (ADCs) are designed to deliver potent cytotoxic agents into tumor tissues. During the last two decades, a plethora of ADCs have been successfully developed and used for several indications, including hematologic and solid tumors. In this work, we systematically reviewed the progress in ADC development for the treatment of HNC. Methods: This review was registered in PROSPERO database. A comprehensive search was conducted following PRISMA guidelines and using PubMed, Scopus and Web of Science database. Results: In total, 19 studies were included. Due to the significant heterogeneity of the outcome measures, meta-analysis was not performed, and data were summarized in tables. HNC results are poorly represented in the cohorts of completed clinical trials; published data are mostly focused on safety evaluation rather than efficacy of ADCs. Conclusions: Although several novel agents against a wide range of different antigens were investigated, showing promising results at a preclinical level, most of the targets reported in this review are not specific for HNC; hence, the development of ADCs tailored for the HNC phenotype could open up new therapeutic perspectives. Moreover, the results from the present systematic review call attention to how limited is the application of current clinical trials in HNC.

## 1. Introduction

Globally, head and neck cancer (HNC) is the sixth most common cause of cancer-related deaths and includes a wide group of tumors that can involve the oral cavity, oropharynx, nasal cavity, paranasal sinuses, nasopharynx, larynx and hypopharynx, accounting for over 600,000 new cases diagnosed per year [1]. About 90% of all HNCs are squamous cell carcinomas (SCCs), which mostly affect the oral cavity [2]. Despite improvements in the knowledge of this disease and its treatment options, patients are still characterized by a 5-year survival rate of about 65%, one of the lowest in all major human cancers [3]. If detected and treated early (Stages I and II), patients might expect a 5-year survival rate higher than 80%, and radiation therapy (RT) or surgical resection are usually curative [4]. However, about two-thirds of patients are initially diagnosed with local and regionally advanced disease (Stages III or IV), and their 5-year survival rate drops to about 65% and 40%, respectively [5].

So far, surgical removal represents the first-line treatment for HNC, complemented by chemotherapy, including cisplatin (CDDP), 5-fluorouracil (5-FU) and docetaxel [6,7], and/or radiotherapy (RT), particularly at advanced stages [8]. For patients with recurrent or metastatic (R/M) disease, treatment is generally multimodal, consisting of surgery followed by chemoradiotherapy, and, despite recent advances, response rates have not been able to demonstrate an improvement in overall survival (OS), which still ranges from 6 to 9 months (3 months for refractory patients) [9,10].

Moreover, these current conventional HNC therapies lack selectivity for cancer cells, leading to systemic toxicity with heightened risk for both acute and long-term adverse effects, and, in addition, evidence of drug-resistant tumors is emerging [11,12].

Therefore, there is an urgent need to investigate the potential of new therapies for HNC. In the last 20 years, only two new targeted therapeutics have been approved for clinical use [13]: cetuximab-based immunotherapy in 2006 [14] and nivolumab- and pembrolizumab-based immunotherapy in 2016 [15] and 2017 [16], respectively. Cetuximab is a chimeric monoclonal antibody (mAb) that selectively binds to the epidermal growth factor receptor (EGFR), inhibiting its role in cancer-cell proliferation, resistance to apoptosis and metastasis [17]. Although its effectiveness against loco-regionally advanced HNC or R/M HNC was reported in various clinical studies [6,18,19,20], some patients still may not benefit from this treatment [21,22], which showed efficacy in only 10–20% of cases [23]. In addition, Petrelli et al. [24], in a meta-analysis of 15 trials, showed that conventional CT [25] was associated with better survival outcomes at two years compared to the administration of cetuximab together with RT. In the last instance, cetuximab was administered together with conventional CT but did not result in improved patients’ OS, leading to an increase in adverse events [26]. On the other side, even though both nivolumab and pembrolizumab have resulted in increased survival and lower severe adverse effects [25], not everyone can benefit from these treatments, and some patients’ results showed resistance to immunotherapy [27]. In this complex scenario, there are still different issues to overcome, such as toxicity, safety, resistance, and efficacy.

Antibody-drug conjugates (ADCs) have established themselves in the therapeutic landscape as the potential answer to this primary oncologists’ demand, taking advantage of the high specificity of mAb to deliver targeted potent chemotherapeutic payloads only to cancer cells [28,29]. This tumor-directed delivery system is designed to reduce off-target toxicities in patients by limiting the exposure of normal tissues to the active cytotoxic component [30]. Since the first ADC was introduced in clinical practice by Food and Drug Administration (FDA) in 2009 [31], to date only 10 ADCs have been approved, 7 of which were approved between 2018 and 2021 [32,33]. Most of these new agents showed efficacy in the treatment of hematological cancers, such as acute myelogenous leukemia [34], anaplastic large cell lymphoma [35], lymphoblastic leukemia [36], diffuse large B-cell lymphoma [33,37], multiple myeloma [38] and hairy cell leukemia [39], but interesting results have been also obtained for solid cancers, with improved outcomes in breast [40,41,42] and urothelial cancer [43]. However, so far, there are no FDA-approved ADCs for HNC treatment, although several potential targets are under investigation in different preclinical/clinical studies. Recently, cetuximab sarotalocan has been approved in Japan for the treatment of unresectable locally advanced and recurrent HNC [44]. Therefore, we aim to systematically review the current evidence focusing on ADCs as potential therapeutics in HNC, summarizing the distinct components of ADCs, including the target antigen, the antibody construct, the linker technology for the payload and the payload itself, its effects and issues, to find opportunities for future avenues of research.

## 2. Materials and Methods

This systematic review was performed according to the guidelines of the Preferred Reporting Items for Systematic Reviews and Meta-analyses (PRISMA) statement [45,46]. The review record has been approved by the international prospective register of systematic reviews PROSPERO under the identification number CRD42021235136.

### 2.1. Search Strategy

An electronic search on scientific databases (PubMed, Scopus, Web of Science) was performed to identify suitable studies, published after 31 December 1999, using the following terms and keywords alone or in combination: (“head and neck cancer” OR “oral cancer” OR oropharynx OR larynx OR nasopharynx OR thyroid OR nose OR “salivary glands”) AND (Immunoconjugate OR “Antibody-Drug Conjugates” OR “Antibody Drug Conjugate” OR Radioimmunoconjugates). The first search was performed on 18 January 2021. The search was limited to studies published in the English language. The last electronic search was performed on 16 May 2021. In addition to the electronic search, a hand search was undertaken by checking the references of the included studies and of the main systematic reviews on molecular targeted therapy in HNC to identify further eligible studies. A reference manager software program (Endnote X9.3.2, Clarivate Analytics) was used, and the duplicates were discarded first electronically, then by checking the resulting list manually.

### 2.2. Eligibility Criteria

#### 2.2.1. Inclusion Criteria

Clinical prospective studies with a cross-sectional, cohort and case-control design were included, as well as preclinical studies, both in vitro and on animal models. Patients included in the study had to be over 18 years old, be diagnosed with HNC and treated with ADC. Clinical outcomes had to be reported, such as efficacy and safety. For preclinical studies, in vitro and in vivo assays had to performed on xenografts derived from HNC cell lines or patient-derived xenografts (PDX).

#### 2.2.2. Exclusion Criteria

Case reports or series, systematic review, meta-analyses, and ecologic studies, as well as studies performed on patients with thyroid cancer or with a histological diagnosis different from SCC; articles assessing ADCs for diagnostic purposes (radioimmunoconjugates) or in combination with antisense approaches or conjugated with bacterial toxins were excluded.

### 2.3. Focused PICO Question

Participants: patients with diagnosed HNC, as well as HNC cell lines or xenograft models.

Intervention: documented protocol of ADC administration.

Comparison: the studied model (cell line, xenograft, or human) exposed to other kind of CT treatments or different ADC protocols.

Outcomes: primary outcomes were safety and pharmacokinetic profile (PK), while secondary outcomes included in vivo efficacy.

### 2.4. Selection of Studies

Retrieved citations were independently screened by two authors (VCAC and VP), and relevant studies were identified based on title and abstract. If those did not provide sufficient information about the inclusion criteria, the full text was evaluated to assess eligibility. Any disagreement was solved by discussion, and a third reviewer was consulted to make final decisions (GS). This author also calculated a k-statistic value to ascertain the level of reviewers’ agreement. In case of multiple publications of the same patient population, only the one with the longest follow-up period was referred to in the text, and the others were considered for data analysis.

### 2.5. Data Extraction and Method of Analysis

Two reviewers (VCAC and VP) independently extracted data from all the included studies using a predesigned extraction form. Microsoft Excel 2020 (Microsoft Office, Microsoft Corporation, Redmond, WA, USA) was used for data collection and for descriptive analysis. The following data were collected: ADC agent name and trade name if present, antigen target, payload, linker type, type of cancer target, phase, sample size, study population/model, ECOG status, primary outcomes, secondary outcomes, developer, references, and notes. The primary outcomes included the safety and efficacy of ADC treatment in clinical studies and cytotoxicity assessment for in vitro/vivo-xenograft models. When applicable, pharmacokinetics and pharmacodynamics were extracted as secondary outcomes. Safety was based on the number and severity of adverse events and maximum tolerated dose; efficacy was based on instrumental evidence of decrease of cancer or remission. For preclinical studies, measures concern cytotoxicity assay as mean difference of cells still alive after ADC exposure among groups or difference in tumor dimension means in xenograft models.

### 2.6. Risk of Bias Assessment

The risk of bias of the included studies was independently assessed by two reviewers (VCAC and VP), as part of the data extraction procedure. The Newcastle-Ottawa scale was applied for in vitro studies, while SYRCLE [47] and RECIST 1.1 [48] were used respectively for in vivo tumors, xenograft models and clinical trials of patients [49]. Each item was defined as adequate when meeting the specific criteria (low risk of bias) and as inadequate (high risk of bias) or n/a when not evaluable. According to this risk of bias assessment method, various items were evaluated. Any disagreement was solved by discussion or consulting a third reviewer (GS) until consensus was achieved.

### 2.7. Statistical Analysis

Considerable methodological and clinical heterogeneity was found in the selected studies regarding participants, interventions, and outcome measures. Evident differences were seen in study design, methodologies, treatment modalities, outcome measures and results. Moreover, many studies reported aggregated results with only descriptive or graphic representation of HNC-related outcomes and without a standardized system. Finally, only a few studies performed statistical analysis of data; therefore, comparisons between studies were not feasible and a descriptive presentation had to be adopted, since meta-analysis was considered inappropriate [50]. However, when possible, quantitative data were presented. Moreover, when applicable, based on each study categorization and criteria, descriptive results were arranged ordinally, and comparisons were made within each factor.

## 3. Results

### 3.1. Study Characteristics

**Figure 1:**
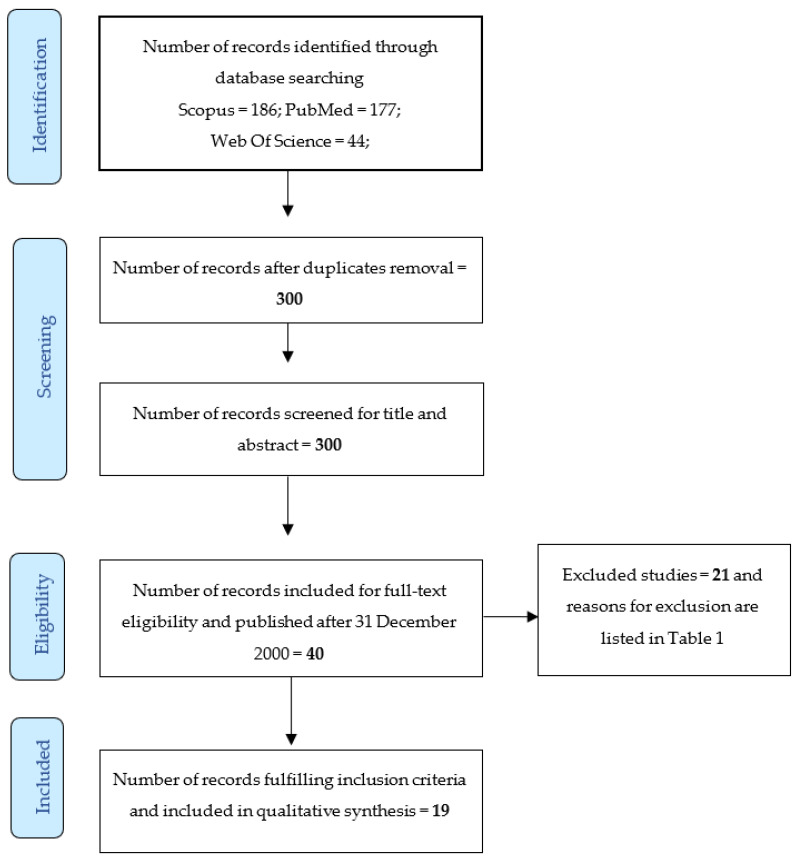
PRISMA 2009 flow diagram of the screening process. In total, 19 studies [51,52,53,54,55,56,57,58,59,60,61,62,63,64,65,66,67,68,69] were included in the present systematic review.

In total, 19 studies [51,52,53,54,55,56,57,58,59,60,61,62,63,64,65,66,67,68,69] were included in the present systematic review (Figure 1).

Twenty-one articles [70,71,72,73,74,75,76,77,78,79,80,81,82,83,84,85,86,87,88,89,90] were excluded after full-text evaluation (Table 1). The k-statistic value was 0.8134, which indicates an excellent level of agreement between reviewers. 

Based on development status, the studies were categorized into 2 main groups:

- Thirteen [51,52,55,56,57,58,60,61,64,65,66,67,69] out of 19 articles investigated different ADCs at a preclinical stage (Table 2);
- Six studies [53,54,59,62,63,68] investigated different ADCs in phase I or II clinical trials (Table 3).

### 3.2. Risk of Bias

The Newcastle-Ottawa scale showed an overall low-risk of bias for all the 13 studies performed on cell line models [51,52,55,56,57,58,60,61,64,65,66,67,69]. Based on SYRCLE features, studies resulted in a high risk of bias. Specifically, only five studies [56,64,65,67,69] reported a sequence generation for the allocation of mice based on randomization. Mice baseline characteristics were reported in few included studies [51,52,56,60,61,66]. Five items of the SYRCLE assessment tool—“allocation concealment”, “random housing”, “blinding of caregivers”, “blinding of assessors” and “incomplete outcome data”—contributed to a high risk of bias, which was inadequate in all of the included studies. Both “random outcome assessment” and “selective outcome reporting” results were adequate in all of the included studies. When considering clinical studies, RECIST v1.1 was used to assess the risk of bias. Evaluation included seven items. In particular, five [53,54,59,62,68] out of six studies reported a statement in which authors followed RECIST guidelines. Baseline measurements were adequately reported in all studies, except for Sauter et al. [63]. When illustrating methods of assessment, such as CT or magnetic resonance imaging, Ocean et al. [59] did not specify the mean. In 4 studies [53,54,62,68], methods of assessment were correctly reported. Assessment of overall tumor burden was not clearly indicated in some manuscripts, and, as a consequence, these were evaluated as inadequate [53,54,59,62,63,68]. Response criteria results were adequate in all included studies; moreover, five [53,54,59,62,68] out of six studies clearly reported the evaluation of best overall response and frequency of tumor re-evaluation. Sauter et al. [63] did not investigate tumor response, and, for this reason, RECIST guidelines were not followed by the authors.

### 3.3. Preclinical Studies

In 13 studies [51,52,55,56,57,58,60,61,64,65,66,67,69], ADCs were investigated in a preclinical stage. FaDu represented the most-used cell line in the included studies [51,56,60,65,66,69], and the cytotoxicity of the ADC was the most-assessed outcome [51,52,55,56,57,58,60,64,65,66,67,69]. Seven [51,56,58,61,64,66,67] out of 13 preclinical studies presented in vivo efficacy outcomes in HNC PDX models. EGFR was the ADC target in two studies [51,69] and HER-2 in one study [55]. Pyrrolobenzodiazepine (PBD) was used as the payload in three studies [51,56,58], whilst monomethyl auristatin E (MMAE) was used in two [61,67]. Non-cleavable linker was used in only two studies [61,66]. Because of the high heterogeneity of study designs, models and investigation, here we summarize findings for each included study.

#### Anderson et al., 2020

ABBV-321 is a next-generation EGFR-targeted ADC that incorporates a PBD dimer cytotoxic molecule conjugated to the EGFR-targeting ABT-806 affinity matured AM1 antibody. Deregulated EGFR activity has been linked to the development, progression, and metastasis of HNC. Furthermore, EGFR overexpression may represent a prognostic marker correlating with decreased radiation sensitivity and increased risk of recurrence [91]. EGFR amplification is the main mechanism leading to its overexpression, and it is present in 10–15% of HNC [92].

Like its predecessors, depatux-m and ABBV-221, ABBV-321 binds a cryptic EGFR epitope located on the cell surface, exposed primarily in tumors when the receptor is activated, thereby providing tumor selectivity. ABBV-321 is differentiated from earlier generation ADCs in that it targets a wide array of tumors beyond those with high levels of EGFR overexpression or amplification, including ones insensitive to auristatin-based ADCs. ABBV-321 administered at a single dose of 0.3 mg/kg was highly effective in inducing sustained regression in A253-derived xenografts (EGFR IHC score of 2+) and a complete regression of FaDu-derived xenografts (EGFR IHC score of 3+) after 45 days. Moreover, five HNC patient-derived xenograft mouse models (CTG-505, CTG-152, CTG-149, CTG-786, CTG-434) treated with 0.6 mg/kg/day ABBV-321 Q7Dx6 showed tumor growth inhibition (TGI_max_ %) ranging between 78–96% and tumor growth delay (TGD %) in the range of >82–>560.

#### Gymnopoulos et al., 2020

TR1801-ADC is a novel cMet-targeted third-generation ADC, site-specifically conjugated to the PBD toxin–linker tesirine (SGD-3249). In HNC specimens, c-Met was overexpressed in 84% of cases, with a nuclear localization mainly at the invasive front [93]. Furthermore, c-Met overexpression correlates with lymph-node metastasis, pathologic stage and disease reoccurrence [94]. Tesirine shows reduced hydrophobicity and the same potency as talirine (SGD-1910) [95]. Increasing the hydrophilicity of the payload potentially leads to a better-tolerated toxin by reducing off-target toxicity [96,97]. Ten HNC PDX models with various levels of cMet expression were treated with a single intravenous administration of the vehicle (PBS), TR1801-ADC (1, 0.5, 0.25, and 0.125 mg/kg) or non-targeting ADC (1 mg/kg). Complete tumor regression was observed in 30% (3/10) of HNC PDX models when treated with a single dose of 1 mg/kg-1TR1801-ADC. Fifty percent (5/10) of the models showed partial regression, and two models showed no significant anti-tumor activity. The three models with complete tumor regression were analyzed by IHC with rabbit monoclonal cMet antibody (SP44), and its expression ranged between high (HN3533 H-score = 300), medium-high (HN0696 H-score = 180) and medium (HN0635 H-score = 130).

#### Scribner et al., 2020

MGC018 is comprised of the cleavable linker-duocarmycin payload, valine-citrulline-seco duocarmycin hydroxy-benzamide azaindole (vc-seco-DUBA), conjugated to an anti-B7-H3 humanized IgG1/kappa mAb through reduced interchain disulfides, with an average drug-to-antibody ratio (DAR) of approximately 2.7. B7-H3 is an immune checkpoint protein and an important regulator of the adaptive immune response [98]. B7-H3 seems to be overexpressed in different HNC subtypes, and its high expression is an adverse prognostic factor for distant control and tumor-specific survival in HNC [99]. HNC PDX model (H score: 240) treated with MGC018 at 3 mg/kg (Q2W × 2) led to 98% reduction in tumor volume compared with vehicle-treated animals, while only 52% tumor reduction was detected in mice treated with control ADC at 3 mg/kg.

#### Ghanemi et al., 2018

Idarubicin-ZHER2 conjugate is an affibody constructed using sulfo-SMCC (a heterobifunctional crosslinker). Affibody molecules represent potential new tumor-targeting agents, composed of three alpha helices with 58 amino acid residues and a molar mass of about 6.5 kDa. Idarubicin-affibody ZHER2:342 conjugate, due to the expression of HER2 receptors on HN5 cells, could reduce the viability of these cells to a maximum of 40%. The in vitro cytotoxicity of the conjugate was lower than the free idarubicin, and it was not possible to increase the concentration of conjugates to compensate for its lower efficacy.

#### Purcell et al., 2018

ABBV-085 is directed against the membrane protein leucine-rich repeat containing 15 (LRRC15), composed of an anti-LRRC15 humanized IgG1 antibody (Ab1) conjugated to monomethyl auristatin E via a protease cleavable valine-citrulline (vc) linker. ABBV-085’s unique mechanism of action relied upon the ability of LRRC15-specific mAb to localize the MMAE payload at high levels in the TME and the cell-permeable properties of MMAE to diffuse into nearby cancer cells and ultimately, induce tumor shrinkage. ABBV-085 used at 12 mg/kg in combination with RT showed enhanced activity in the HNC xenograft model SCC15 (stromal-positive only, 3+ IHC), which was statistically superior to either agent alone. The combination activity with ABBV-085 resulted in 33% complete response (CR) and 67% partial response (PR).

#### Theunissen et al., 2018

The aim of this preclinical study was to develop tissue factor (TF/CD142)-specific ADC with no impact on the coagulation cascade, an adverse event typically associated to the use of anti-TF ADCs. TF is a transmembrane protein receptor and the main initiator of the extrinsic coagulation cascade, leading to fibrin formation. Only one study explored the expression of TF in HNC, showing a highly tumor-specific expression pattern [100]. Researchers identified one paratope family of antibodies, which did not affect blood clotting, and conjugated them to the prototypic cytotoxic agent MMAE through a protease-cleavable linker. A HNC PDX model with a H-score 250 was treated with a dose of 5 mg/kg (QWK × 2), and, at day 60, in the group treated with the prototype ADC, named 25A-vc-MMAE, 5 out of 10 animals showed a partial regression, and 1 animal was tumor-free, while, in the group treated with 43Ea-vc-MMAE, 6 out of 10 animals showed a partial regression.

#### Wong et al., 2018

RN765C is composed of a low-affinity anti-EGFR hIgG1, conjugated with PF-06380101, a potent antimitotic agent, through the cleavable AcLys-VC-PABC linker. The peculiar low affinity enables the antibody to bind only transiently to its target on normal tissue wherein the EGFR density is low compared to EGFR-overexpressing tumor cells, minimizing adverse effects. The sensitivity of low-affinity ADC RN765C was evaluated in a panel of EGFR-expressing tumor cells, resulting to be higher in FaDu cells (Ec = 0.806 nM) compared to normal human epidermal keratinocytes.

#### Kerk et al., 2017

MEDI0641 targets the oncofetal antigen (5T4, known also as trophoblast glycoprotein), a transmembrane protein involved in several cellular functions, such as epithelial-to-mesenchymal transition. Furthermore, it is a potential marker for cancer stem cells (CSCs), is specific to HNC treatment [101] and carries PBD as its DNA-damaging payload. PBD dimer is released in cancer cells through the proteolytic cleavage of the Val-Ala dipeptide and following the self-immolation of the PABA spacer at the low pH of the lysosomal compartment. In vitro, MEDI0641 caused a significant reduction in the CSC fraction in HNC cells, while in vivo, its therapeutic efficacy was examined in three PDX models expressing 5T4 generated from different clinical settings: PDX-SCC-M0, PDX-SCC-M1 and PDX-SCC-M11. A single dose of MEDI0641 (0.33 mg/kg) resulted in complete tumor regression in the PDX-SCC-M0 model with a prolonged survival up to 150 days after treatment. In a second setting, MEDI0641 antitumor activity was evaluated in PDX-SCC-M1 and PDX-SCC- M11 at a dose of 1 mg/kg, resulting in significant regression in tumor volume in both models.

Additionally, various treatment regimens were evaluated in the PDX-SCC-M11 model (a single dose of 1 mg/kg, 1 dose of 0.5 mg/kg every three weeks or 1 dose of 0.33 mg/kg every three weeks), and all three regimens caused a significant reduction in tumor volume for at least 100 days. Finally, MEDI0641 has proven to be able to prevent local recurrence in vivo by treating PDX-SCC-M11 with either 1 mg/kg IgG1-PBD control or 1 mg/kg MEDI0641 before the surgical removal of the tumor, and no recurrences were observed in 12 mice in the MEDI0641-treated group, whereas the IgG1-PBD control group showed recurrences in 7 of 12 mice.

#### Strop et al., 2016

RN927C is a cleavable anti-Trop-2 ADC developed using a site-specific transglutaminase-mediated conjugation method and a proprietary microtubule inhibitor linker-payload, PF-06380101, able to, upon binding to the extracellular portion of Trop-2 on the cancer cell surface, be internalized and induce cell-cycle arrest, resulting in cell death. RN927C efficacy data on HNC are limited to in vitro cytotoxicity on FaDu cells resulting in IC50 of 0.507 ± 0.219 nM.

#### Sweeny et al., 2013

EDC22 is an extracellular drug conjugant targeting CD147, linked to a small drug molecule inhibitor of Na/K-ATPase. CD147 is a transmembrane glycoprotein highly expressed on the cell membrane of HNC cells, adjacent to the Na/K-ATPase as part of a larger complex [102], so the delivery of Na/K-ATPase inhibitor linked to an anti-CD147 mAb may result in a highly toxic chemotherapeutic effect. CD147 plays an important role in HNC tumorigenesis and progression by promoting epithelial-to-mesenchymal transition [103]. Furthermore, its inhibition enhances chemosensitivity in HNC cells by inhibiting the MAPK/ERK signaling pathway [104]. The in vitro treatment of HNC cell lines (SCC-1, OSC-19, FaDu and Cal27) with EDC22 significantly reduced proliferation. EDC22’s in vivo antitumor efficacy was evaluated in an OSC-19 orthotopic model using as regimens 3 mg/kg/wk or 10 mg/kg/wk systemically for 18 days, plus an additional arm with 30 mg/kg/wk of naked anti-CD147 antibody. Relative to control and naked anti-CD147 groups, for both EDC22 treatment cohorts, a reduction in tumor growth was observed, with no advantage at the higher dosage of EDC22 (10 mg/kg). In vivo treatment with EDC22 (3 mg/kg/biweekly) systemically for 18 days was evaluated in a SCC-1 subcutaneous xenograft model compared to cisplatin and radiotherapy. There was a significant reduction in tumor growth with EDC22 compared to cisplatin monotherapy or RT, with no advantage in combination treatment.

#### Chen et al., 2012

HLEAFab-MMC is an immunoconjugate directed against the LMP1 extracellular domain containing mitomycin C (MMC) as the payload. LMP1 is considered to be the major oncoprotein of Epstein–Barr virus (EBV), a herpes virus prevalently associated with a variety of malignant diseases, such as nasopharyngeal carcinoma (NPC).

To determine the efficacy of HLEAFab-MMC to specifically inhibit the growth of HNE2/LMP1cells, in vitro proliferation and apoptosis assays were carried out. Results showed that HLEAFab-MMC was effective in inhibiting 76% of cell proliferation at 200 nmol/L compared to HNE2 control cells, wherein the ADC exhibited very modest or no cytotoxicity. In addition, as for proapoptotic activity, the highest apoptosis rate in HNE2/LMP1 cell lines was observed in the HLEAFab-MMC group (13.88%) compared with the control groups. Finally, mice models bearing HNE2/LMP1 xenografts were established, and mice were treated on days 1, 4 and 7 with HLEAFab-MMC (1.6 × 10^5^ mol/kg), MMC equivalent at 0.053 mg/kg and HLEAFab equivalent at 8 mg/kg. ADC-treated mice achieved an average 55.1% inhibition of tumor growth in comparison with their controls.

#### Osterman et al., 2008

Novel antibody-maytansinoid conjugates FAP5-SPP-DM1, FAP5-SPDB-DM4 and FAP5-SMCC-DM1 were developed in this research study to target a shared epitope of human, mouse and cynomolgus monkey fibroblast activation protein-a (FAP alpha), enabling preclinical efficacy and tolerability assessments. FAP alpha overexpression on cancer-associated fibroblasts has been confirmed in more than 90% of epithelial carcinomas, including HNC, while it is nearly absent in normal adult tissues [105]. FAP5-SPP-DM1 and FAP5-SPDB-DM4 are based on the use of two analogues of maytansinoids DM1 and DM4 linked to the mAb via a cleavable linker, while in FAP5-SMCC-DM1 a non-cleavable linker was used. A Fadu xenograft model was treated with a single dose of 600 mg/kg of the three ADCs or the vehicle, and a prominent antitumor effect was observed in FAP5-SPP-DM1 and FAP5-SPDB-DM4 groups, including complete tumor regressions in three out of six animals. After the discontinuation of therapy (days 20–27), a large proportion of animals remained free of palpable tumors or showed no further growth during an extended observation period (3–4 weeks).

#### Herbert et al., 2003

SPA470-doxorubicin is directed against Hsp47/CBP2, a heat-inducible glycoprotein that seems to be involved in HNC progression by mediating the endogenous processing of collagen XVIII in tumor cells [106]. In vitro cytotoxicity was evaluated in SCC15 cells using colony formation assay, and the results revealed that treatment with SPA470-DOX for 2 h was significantly more potent than unconjugated DOX, DOX-hydrazone or equivalent mAb protein + DOX. The observation that SPA470-DOX is effective during hypoxia or conditions that mimic hypoxia presumes the further utility of SPA470-DOX in treating head and neck cancers, because pO_2_ values below 2.5 mmHg are a common feature in human oral squamous carcinoma cells from patients with such malignancies.

### 3.4. Clinical Studies

In six studies [53,54,59,62,63,68] ADCs were investigated in phase I or II clinical trials. Safety of the ADC was the most assessed outcome. Five [53,54,59,62,68] out of six clinical studies presented in vivo efficacy outcomes, but the endpoints were heterogeneous and measured at different time-points. ADC targets were different among all studies. MMAE has been used as a payload in two studies [53,54], and cleavable linkers were used in all the studies. Forty-seven patients affected by HNC over a total of 331 patients were treated in the clinical studies. Only two studies [62,63] evaluated the ADC in a cohort of 31 HNC patients.

#### Cleary et al., 2020

Losatuxizumab vedotin (formerly ABBV-221) is a second-generation ADC targeting EGFR, consisting of depatuxizumab, a cathepsin-cleavable valine-citrulline (vc) linker and a potent microtubule polymerization inhibitor, MMAE. Only 5 out of 45 enrolled patients were affected by HNC cancer; however, the demographics cannot be extracted as the data are aggregated. The maximum tolerated dose (MTD) was not reached due to infusion-related reactions (>45%); other treatment-emergent adverse events (TEAEs) were represented by fatigue, diarrhea, nausea, rash and pruritus. Moreover, all 45 patients discontinued the study; the most common reason was progressive disease, and no patients achieved a complete remission. Stable disease for at least two cycles was observed in one HNC patient (EGFR +/EGF −) remaining in the study for >6 months, and partial remission was achieved in another patient (EGFR +/EGF +) previously treated with cetuximab, with five cycles (15 weeks) of losatuxizumab vedotin.

#### Tsurutani et al., 2020

Trastuzumab deruxtecan (T-DXd; DS-8201a) is a novel ADC with a humanized anti-HER2 antibody, a cleavable peptide-based linker and a potent topoisomerase I inhibitor payload. A dose of 6.4 mg/kg every three weeks was chosen for dose expansion in the cohort named “other cancers” that included eight cases of salivary-gland tumors. The patients’ demographics and baseline characteristics, as well as the in vivo efficacy endpoints of the enrolled HNC, cannot be extracted as the data are aggregated. Authors specified that in the other cancer group, salivary duct carcinoma had the most pronounced shrinkage, although only a reduction in tumor size of about 20% was shown.

#### de Bono et al., 2019

Tisotumab vedotin is a first-in-human ADC directed against tissue factor and conjugated to the microtubule-disrupting agent MMAE via a protease-cleavable valine-citrulline linker. One HNC patient was enrolled in the dose-escalation phase in 0.6 mg/kg dose cohort, and he died from a pharyngeal tumor hemorrhage.

#### Ocean et al., 2017

The authors developed an anti-Trop-2 ADC (sacituzumab govitecan/IMMU-132) based on the well-known anticancer drug SN-38, a camptothecin that is the active component of irinotecan (CPT-11), a topoisomerase I inhibitor. Only two patients affected by HNC out of 178 were enrolled, and only data about safety and tolerability, as well as adverse events, were reported in relation to the administered doses.

#### Riechelmann et al., 2008

BIWI 1 (bivatuzumab mertansine) is an immunoconjugate consisting of DM1, a highly potent antimicrotubule agent coupled to a IgG_1_ kappa mAb against CD44v6, which is strongly expressed by more than 95% of HNC, mostly pharyngeal and laryngeal cancers, and is related to advanced stage and poor prognosis [107]. However, it is not tumor-selective as it is also expressed on normal squamous epithelium, including skin keratinocytes. Binding to CD44v6 on skin keratinocytes mediated serious skin toxicity, with almost 80% of the patients experiencing adverse events related to skin and their frequency and intensity increased with increasing doses with a fatal outcome in a parallel trial, which led to the termination of the development program of bivatuzumab mertansine and the present study. The only available data are related to the 10% response rate, in three patients at doses of 200, 275 and 325 mg/m^2^; tumor regression lasted 4–8 months, and the disease progressed again upon suspension of the treatment. Median TTP was 79 days, ranging from 17–385 days.

#### Sauter et al., 2006

In this dose escalation phase I trial, BIWI 1 was investigated with an explanatory nature, and preliminary safety, immunogenicity and PK profile analyses were conducted. The main toxicity of ADC determining the MTD of 300 mg/m^2^ was related to skin, which can be explained by the binding of the antibody component bivatuzumab to keratinocytes. No positive anti-BIWI 1 immune response either after single or multiple dosing was observed; therefore, the data suggest that bivatuzumab mertansine has no or only very little immunogenicity, at least after short-term administration. The PK profile of bivatuzumab mertansine corresponded well to the known PK behavior of other humanized mAbs.

### 3.5. Clinical Trials

The last search was performed on 16 May 2021 on https://clinicaltrials.gov/ (accessed on 16th May, 2021) using “Head and neck cancer” or “Head and neck squamous cell carcinoma” as inputs in “condition” field, while “other terms” was filled with “Antibody-Drug Conjugates”. No restrictions were applied in “Country”. Ten studies were displayed. One clinical trial, NCT02465060, was excluded for not dealing with ADCs. The results of two clinical trials (NCT01631552 and NCT02001623) were already reported in the studies of Ocean et al. [59] and de Bono et al. [54], respectively. An additional trial, NCT03234712/I, was found through a manual search. The results of the retrieved eight clinical trials have not been published yet. Details about the name of the investigated ADC, target, payload, linker, weblink, clinical trial code, sponsor and status are reported in Table 4. For the clinical trial NCT03602079, the payload has not been disclosed so far; therefore, not specified (NS) was reported in the table.

## 4. Discussion

HNC is a broad term that encompasses epithelial malignancies that arise in the paranasal sinuses, nasal cavity, oral cavity, pharynx, and larynx. The standard of care for HNC involves a combination of surgery, radiation and chemotherapy, but at least 50% of initially treated patients develop R/M within two years [108]. The current therapy for R/M HNC is first-line platinum CT in combination with 5-FU and cetuximab, which resulted in a median overall survival of 10.1 months [19]. Recently, the importance of the immune system in the development and treatment of HNC has long been recognized, leading to the FDA approval of immune checkpoint inhibitors in 2016–2017 (nivolumab and pembrolizumab) for the treatment of R/M disease and for front-line treatment of inoperable or metastatic cancer in 2019, definitively expanding the landscape of HNC therapy. Nevertheless, to date, only a subset of patients showed long-term benefit with immune checkpoint inhibitors, and reliable predictive biomarkers are needed [1]. It is rather evident that safer and more effective therapies are urgently needed for HNC patients, because treatment approaches based only on surgery, RT, CT and biotherapeutic antibodies fail to reach satisfactory results in terms both of survival and quality of life for HNC patients. Particularly, there has been a great expectation for antibodies conjugated to potent cytotoxic agents (ADCs) or gold nanoparticles (GNPs) [109]. In this review, we focused on the development of the ADCs at the preclinical and clinical levels.

The development of this class of agents is challenging as all the three key elements defining the ADC may play a critical role in determining their efficacy and tolerability: the mAb targeting a specific tumor or stroma antigens, the potent cytotoxic drug and the linker connecting the payload to the antibody. Indeed, despite the enormous efforts of the last twenty years and dozens of different novel ADCs generated so far, only 10 of them entered into clinical practice, 3 of which are FDA-approved for the treatment of solid cancers and the remainder for blood malignancies (Table 5).

However, clinical benefits obtained by approved ADCs and the substantial technological advancements allowing the fast improvement of ADC designs, combined with the availability of novel targets coming from multi-omics platforms, are now fueling enthusiasm to develop novel candidates. In line with this, the number of ADCs in clinical trials have more than tripled over the past five years, and approximately 80 of them are in clinical development in nearly 600 clinical trials [29].

**Figure 2:**
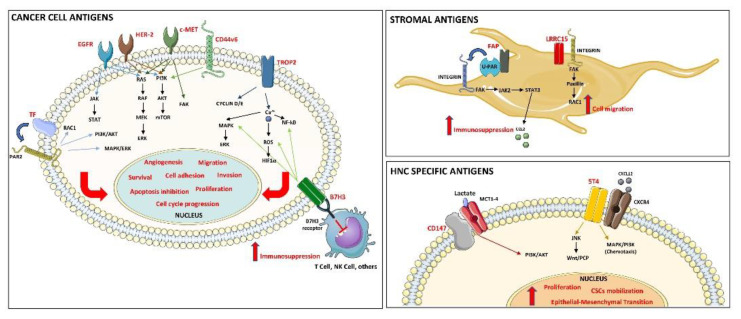
ADC target antigens and the main transduction signaling pathways.

We have reviewed all the research studies on novel ADCs against targets involved in HNC cancer, and their activity in preclinical models and clinical trials has been discussed. At present, because HNC is poorly represented in the cohorts of completed clinical trials, and published data are mostly focused on the safety evaluation of ADCs rather than efficacy, inconclusive information is available to define the clinical relevance of ADCs in HNC therapy. On the other hand, several novel agents against a wide range of different antigens (Figure 2) were investigated, showing promising results in preclinical level in several HNC animal models, including PDX and orthotopic implants. 

Graphical representation of the discussed ADC target antigens and relative main signaling pathways.

Some of these ADC targets are known oncoproteins, such as EGFR (targeted by two ADCs), HER-2, c-MET, Trop-2 or tumor antigens, such as tissue factor (CD142) and B7-H3, the latter being recently validated as a novel immune-checkpoint. All these proteins are found to be overexpressed in cancer cells. Moreover, two novel ADCs were designed to target stroma components: tumor-associated fibroblast antigens LRRC15 and FAP (fibroblast activation protein α).

Among these ADCs, ABBV-321 and TR-1801 (targeting EGFR and c-Met, respectively), have demonstrated robust and promising therapeutic activity in clinically relevant models, such as PDXs, and can therefore be advanced to a more advanced step in the preclinical stage. In particular, ABBV-321, used in a single low dose, exhibited potent anti-tumor activity in five HNC PDXs, and indeed, it is now under investigation in a phase I clinical trial (NCT03234712); also, a single dose of TR-1801 ADC showed complete tumor regression in 30% of HNC PDX models and partial regression in 50% of them. Anti-tissue factor ADCs and MGC018 (targeting B7-H3) were used in a single PDX model, obtaining the partial remission of tumors in both cases; while ADCs targeting fibroblast-associated antigens LRRC15 and FAP, ABBV-085 and FAP5/DM1-DM4, respectively, showed only the tumor-growth inhibition of xenografts derived from commercial established HNC cell lines. It is important to underline that ABBV-085 is in a phase I clinical trial (NCT02565758).

Interestingly, although only in the preclinical stage, two ADCs, MEDI0641 and EDC22, were designed to target antigens specifically expressed in HNC. The first is based on an antibody recognizing 5T4 oncofetal antigen, expressed primarily by CSCs subpopulation of HNC cells. A single injection of MEDI0641 showed long-lasting tumor regression in three PDX models ablating CSC population and the ability to prevent local recurrence in vivo. The second one, EDC22, is a particular type of ADC constituted by a mAb targeting CD147 linked to a small drug molecule inhibitor of Na/K ATPase. Systemic treatment with EDC22 demonstrated significant potency and efficacy in the preclinical treatment of HNC, both in an orthotopic and subcutaneous tumor mouse model; moreover, EDC22 therapy resulted in a greater reduction in tumor growth compared to RT and cisplatin monotherapy. Therefore, the remarkable potency of these ADCs requires further investigations of their clinical potential in the treatment of HNC.

Regarding published clinical studies (Phases I–II), HNC is generally poorly represented in the patient cohorts. The only exception is related to anti-CD44v6 ADC, BIWI 1 (bivatuzumab mertansine), which included 31 HNC patients in both clinical studies [62].

CD44v6 is an intriguing target expressed in more than 95% of HNC patients. However, the expression of CD44v6 is not tumor-selective as it is also expressed on normal squamous epithelium, including skin keratinocytes, leading to serious skin toxicity with a fatal outcome, and the risk–benefit evaluation turned negative for BIWI 1.

Finally, the results of the remaining eight clinical trials have not been published yet. Among them, it is noteworthy to mention three novel agents. One concerns the target CD-71 (transferrin receptor), overexpressed in malignant cells correlated with tumor stage or cancer progression, the object of two clinical trials with CX-2029 and CX-2009 ADCs. The second one tested a particular ADC, SBT6050, comprised of a potent Toll-like receptor 8 (TLR8) agonist conjugated to a HER2-directed mAb, which is immunostimulatory, activating myeloid cells used in HER-2-amplified solid tumors. The third one deals with the clinical development of CD-276, a target that showed promising results in a preclinical study by Scribner (2020) [64].

While intriguing results emerged from preclinical single studies investigating ADCs in HNC, results from the present systematic review call attention to how limited is the application of current clinical trials for HNC. Indeed, only 47 patients affected by HNC were included over a total of 331 patients with solid cancer; and only two studies [62,63] were performed in a cohort of 31 HNC patients. Future research should include a higher number of HNC patients, and, due to the biological heterogeneity of this tumor, they should be classified into more specific subgroups (for example, separating salivary gland tumors from SCCs) [110]. This would make the study cohorts more homogeneous and would help in identifying those subgroups of patients that could potentially attain greater benefits from ADC-based treatments.

The development of effective ADCs for HNCs could benefit from the selection of new targets. Indeed, most of the targets reported in literature are not particularly specific for HNC. In addition to EGFR, several tumor-associated antigens are reported in the literature, particularly expressed in HNC, such as carcinoembryonic antigen (CEA) and mucin 1 (MUC1) [111,112]. The development of more tailored ADCs for the HNC phenotype could open up new therapeutic perspectives. Moreover, the advances in bioinformatics, the development of online databases, such as The Cancer Genomic Atlas (TCGA) and Catalogue of Somatic Mutations in Cancer (COSMIC), and the tumultuous progress of omics in cancer research all improved the practicality of detecting and targeting cancer neoantigens that develop and persist during tumor clonal expansion [113]. Therefore, in the future, it is possible to imagine the development of personalized drugs based on the study of the “mutanome”, defined as the entirety of somatic cancer mutations in an individual tumor [114]. Since angiogenesis exerts a critical role in tumor growth, invasion and metastasis, another attractive therapeutic strategy in solid tumors is anti-angiogenic therapy, based on the target endothelial cells of tumor vessels. The development of ADCs targeting vascular endothelial growth factor receptor (VEGFR) is a promising strategy to obtain an anticancer therapeutic effect without showing tumor resistance [115]. However, to date, none of them has been investigated for the treatment of HNC.

Since most of the studies were performed to address safety and pharmacological properties, maximizing the therapeutic index is the leading prerequisite for the future clinical successful application of ADCs [29]. Another issue that should be addressed is the choice of an eligible preclinical model that could reflect human ADC biodistribution [116], as well as the possibility of evaluating differential target expression among multiple metastases, which are common in patients with R/M disease [29]. At last, successful treatment may be further achieved through the identification of biomarkers, which may inform about patient selection, sensitivity, monitoring and adverse events. To date, only one ADC agent has been approved for patients with HNC. Cetuximab sarotalocan has been approved for clinical use in Japan, and it has shown overall outcome improvements compared to standard therapies [117,118]. Compared to the other recently developed ADCs, cetuximab sarotalocan combined the action of anti-EGFR antibody cetuximab with a payload dye, specifically IRDye 700DX (IR700, LI-COR; Lincoln, NE, USA) near-infrared (690 nm) photosensitizing dye [119].

## 5. Conclusions

HNC is undoubtedly a relevant clinical problem and, despite the partial success achieved with multimodal therapeutic approaches, improving patient survival and quality of life remains a priority. In this work, we systematically reviewed progress in ADC development for the treatment of HNC, specifying the promising targets studied and ongoing clinical trials. In the future, the development of ADCs tailored to the HNC phenotype and clinical trials including cohorts with numerous HNC patients could represent a promising avenue towards novel therapeutic interventions for this multifarious disease.

## Figures and Tables

**Table 1:** List of excluded studies (#21) and reasons for exclusion.

Study	Reasons for Exclusion
Seok et al., 2020 [83]	Anaplastic thyroid carcinoma
Sunavala-Dossabhoy et al., 2020 [85]	Invited commentary
Takei et al., 2020 [86]	mAb
Bera et al., 2019 [72]	Thyroid peroxidase (TPO)-mesothelin (MSLN) mouse model development and antitumor efficacy of LMB-100 (hMSLN-targeted immunotoxin) and anti-CTLA-4 (cytotoxic T-lymphocyte antigen 4)
Mao et al., 2018 [79]	Photodynamic therapy
Zhang et al., 2018 [90]	Near-infrared photoimmunotherapy using gold nanoparticles (AuNPs) conjugated with a mAb targeting the EGFR
Benedetto et al., 2017 [71]	111In-DTPA-cetuximab radioimmunoconjugate preparation
Jang et al., 2017 [75]	Study performed on patients with thyroid cancers
Munasinghe et al., 2017 [80]	Investigation on specific adverse effect on QT interval prolongation
Nagaya et al., 2017 [81]	Near-infrared photoimmunotherapy using anti-CD44 monoclonal antibodies conjugated to the photoabsorber IR700DX
Challita-Eid et al., 2016 [73]	Immunohistochemical expression of Nectin-4
vanDriel et al., 2016 [87]	Photodynamic therapy
Lamberts et al., 2015 [77]	Expression of membrane-bound glycoprotein mesothelin (MSLN) by functional genomic mRNA profiling in 41 tumor types
Bachran et al., 2013 [70]	Cytotoxicity of *Bacillus anthracis* lethal factor (LFn), N-terminal 389 aminoacids of diphtheria toxin (DT389) and human transforming growth factor alpha (TGFalpha) against EGFR-expressing cell line
Zhang et al., 2013 [89]	Immunohistochemical expression of anti-latent membrane protein 1 (LMP1) in the treatment of advanced nasopharyngeal carcinoma (NPC)
Sandstrom et al., 2011 [82]	(67Ga)Ga-NOTA-Bn-NCS-hEGF radioimmunoconjugate for the diagnostic imaging of EGFR-expressing tumors
Maeda et al., 2009 [78]	Effect of sonoporation and anti-EGFR antibody as a drug-delivery system for treating squamous cell carcinoma
El-Sayed et al., 2006 [74]	Photodynamic therapy
Kawakami et al., 2004 [76]	Effect of nitric oxide (NO) inhibiter on IL-13-PE38QQR (*Pseudomonas* exotoxin) cytotoxin-mediated cytotoxicity
Yamamoto et al., 2004 [88]	*Actinobacillus actinomycetemcomitans* cytolethal distending toxin (Cdt-B)
Strome et al., 2002 [84]	IL--4R-PE38KDEL (*Pseudomonas* exotoxin)

**Table 2:** Characteristics of preclinical studies.

ADC	Target Antigen	Payload	Linker Type	Tumor Type (s)	Models	Company	References	Notes
Serclutamab talirine/ABBV-321	EGFR	PBD dimer SGD-1882 with a fixed DAR of 2.0	Cathepsin-cleavable maleimidocaproyl-valine-alanine (MC-Val-Ala) type linker	Colorectal cancer, glioblastoma, HNC, lung cancer, malignant mesothelioma	HNC cell lines: FaDu, A253 HNC PDX models: CTG-505, CTG-152, CTG-149, CTG-786, CTG-434	AbbVie	Anderson 2020 [51]	NCT03234712—https://adc.expert/2MKZSp2 (accessed on May 16th, 2021)
TR1801-ADC/MT-8633	c-Met	PBD toxin-linker tesirine (SG3249)	Cleavable (Val-Ala)	Biliary tract cancer, colon cancer, gastric cancer, HNC, lung cancer	HNC cell lines: Detroit 562, FaDu. Ten HuPrime HNC PDX models, among three specified: HN3533; HN0635; HN0696	Tanabe Research Laboratories USA in collaboration with Open Innovation Partners and MedImmune/AstraZeneca	Gymnopoulos 2020 [56]	TR1801-ADC in patients with tumors that express c-Met | https://clinicaltrials.gov/ct2/show/NCT03859752 (accessed on May 16th, 2021)
MGC018; ANTI-B7-H3 ADC	B7-H3 (CD276)	Synthetic duocarmycin analogs	Cleavable valine-citrulline-seco duocarmycin hydroxy-benzamide azaindole (vc-seco-DUBA)	Breast cancer, HNC, lung cancer, melanoma, ovarian cancer	HNC PDX model: Not specified	MacroGenics, Inc.	Scribner 2020 [64]	
Idarubicin-Z HER2:342	HER 2	Idarubicin	Cleavable	HNC	HNC cell lines: HN5	//	Ghanemi 2018 [55]
Samrotamab vedotin/ABBV-085	LRRC15	MMAE	Protease cleavable Val-cit	Breast cancer, colorectal cancer, gastric cancer, glioblastoma, HNC, lung cancer, melanoma, osteosarcoma, ovarian cancer, pancreatic cancer, pleomorphic undifferentiated sarcoma, testicular cancer	HNC xenograft models: SCC15	AbbVie. S.E., AbbVie. E.D.	Purcell 2018 [61]	NCT02565758—https://clinicaltrials.gov/ct2/show/NCT02565758 (accessed on May 16th, 2021)
Anti-TF ADCs	TF (CD142)	MMAE	Cleavable	gastric cancer, HNC, ovarian cancer	HNC PDX models: not specified	Iconic Therapeutics, Inc.	Theunissen 2018 [67]	
RN765C	EGFR	PF-06380101 (AUR0101) an auristatin microtubule inhibitor (a cytotoxic dolastatin 10 analogue)	AcLys-VC (valine-citruline)-PABC (cleavable linker)	Breast cancer, colorectal cancer, glioblastoma, HNC, lung cancer	HNC cell lines: FADu	Pfizer/Rinat	Wong 2018 [69]	
MEDI0641	5T4	PBD	Cleavable (dipeptide)	HNC	HNC cell lines: UM-SCC-11B, UM-SCC-22B, HNC PDX models: PDX-SCC-M0, PDX-SCC-M1, PDX-SCC-M11	MedImmune LLC	Kerk 2017 [58]
RN927C	Trop-2	PF-06380101 (AUR0101) an auristatin microtubule inhibitor (a cytotoxic Dolastatin 10 analogue)	Cleavable AcLys-VC-PABC	Breast cancer, colon cancer, HNC, lung cancer, ovarian cancer, pancreatic cancer, skin cancer	HNC cell lines: Fadu	Pfizer/Rinat	Strop 2016 [65]	
EDC22	CD147	Na/K-ATPase inhibitor	Non cleavable heterobifunctional linker	HNC	HNC cell lines: FaDu, OSC-19, Cal27, SCC-1 HNC xenograft models: SCC-1HNC orthotopic models: OSC-19	Centrose, LLC	Sweeny 2013 [66]	
HLEAFab-MMC	LMP1	Mytomicin C	Cleavable N-succinimidyl 3-(2-pyridyldithio) propionate (SPDP)	Nasopharyngeal cancer	HNC cell lines: HNE2 and HNE2/LMP1 transfected, HNC xenograft models: HNE2/LMP1 transfected	//	Chen 2012 [52]	
FAP5-SPP-DM1, FAP5-SPDB-DM4, FAP5-SMCC-DM1	FAPα	Maytansinoids DM1/DM4	Cleavable SPP, Cleavable SPDB, Non cleavable SMCC	Colon cancer, fibrosarcoma, HNC, lung cancer, pancreatic cancer	HNC cell lines: FaDu HNC xenograft model: FaDu	ImmunoGen and Oncotest	Ostermann 2008 [60]	
SPA470-doxorubicin	Hsp47/CBP2	doxorubicin	Cleavable acylhydrazone linker	HNC	HNC cell lines: SCC-4, -9, -15 and -25; UMB2/Hsp47 transfected	//	Herbert 2003 [57]	

**B7-H3:** B7 homolog 3; **CD147:** cluster of differentiation 147; **CD276:** cluster of differentiation 276; **c-Met:** tyrosine-protein kinase Met or hepatocyte growth factor receptor (HGFR); **EGFR:** epidermal growth factor receptor; **FAPα:** fibroblast activation protein α; **HER 2:** human epidermal growth factor receptor 2; **Hsp47/CBP2:** heat shock protein 47; **HNC:** head and neck cancer; **LMP1:** Epstein–Barr virus latent membrane protein 1; **LRRC15:** leucine-rich repeat containing 15; **MMAE:** monomethyl auristatin E; **MMC:** mitomycin C; **PBD:** pyrrolobenzodiazepine; **TF:** tissue factor; **5T4:** oncofetal antigen 5T4 or trophoblast glycoprotein; **Trop-2:** trophoblast cell surface antigen 2.

**Table 3:** Characteristics of clinical studies.

ADC	Target Antigen	Payload	Linker Type	Tumor Type(s)	Phase	Sample Size (Total/HNC)	Stage	Primary Outcomes	Secondary Outcomes	Sponsor/ Collaborator	References	Notes
Losatuxizumab vedotin/ABBV-221	EGFR	MMAE	Cleavable (Mc-Val-Cit-PABC)	Breast cancer, colorectal cancer, glioblastoma, HNC, lung cancer, malignant mesothelioma	I	45/5	ECOG: 0–2	Safety (TEAE, MTD, DLT), PK profile	In vivo efficacy (CR, DOR, ORR, OS, PD, PR, SD, TTP), change in ECOG	AbbVie	Cleary 2020 [53]	stopped for high frequency of infusion-related reactions
Trastuzumab deruxtecan (DS-8201A, T-Dxd)	HER2	Camptothecin analog exatecan (DXd; DX-8951 derivative)	Cleavable tetrapeptide linker, Gly-Phe-Leu-Gly (GFLG)	Biliary tract cancer, breast cancer, colorectal cancer, endometrial cancer, lung cancer, salivary glands cancer	I	60/8	ECOG: 0–1	Safety (TEAE), tolerability	In vivo efficacy (CR, DCR, ORR PD, PFS, PR, SD, TTR)	Daiichi Sankyo Inc.	Tsurutani 2020 [68]	NCT03248492—http://adc.expert/2eYaukS (accessed on May, 16th 2021) NCT03734029—https://clinicaltrials.gov/ct2/show/NCT03734029 (accessed on May 16th, 2021) NCT03523585—https://clinicaltrials.gov/ct2/show/NCT03523585 (accessed on May 16th, 2021) NCT03529110—https://clinicaltrials.gov/ct2/show/NCT03529110 (accessed onMay 16th, 2021)
Tisotumab vedotin/TF-011-MMAE/HUMAX-TF-ADC	TF (CD142)	MMAE	Cleavable (Val-Cit)	Bladder cancer, cervix cancer, endometrial cancer, HNC, lung cancer, oesophagus cancer, ovaric cancer	I-II	27/1	ECOG: 0–1	Safety (CTCAE)	MTD, PK profile, in vivo efficacy (CR, DCR, DOR, ORR, PFS, PR, SD)	Genmab/Seattle Genetics	de Bono 2019 [54]
Sacituzumab govitecan/IMMU-132/HRS7-SN38	Trop-2	Camptothecin analog (SN38) Irinotecan metabolite 7-ethyl-10 hydroxycamptothecin	Cleavable carbonate	Bladder cancer, colorectal cancer, gastrointestinal cancer, HNC, kidney cancer, lung cancer, ovaric cancer, pancreas cancer, prostate cancer	I-II	178/2	ECOG: 0–1	Safety (CTCAE), PK profile	In vivo efficacy	Immunomedics	Ocean 2017 [59]	NCT01631552—https://clinicaltrials.gov/ct2/show/NCT01631552 (accessed on May 16th, 2021) NCT02161679—https://clinicaltrials.gov/ct2/show/NCT02161679 (accessed on May 16th, 2021)
Bivatuzumab mertansine/BIWI-1	CD44v6	DM1	Cleavable disulfide	HNC	I	31/31	ECOG: 0–2	Safety (CTC, DLT, MTD,) PK profile	*In vivo* efficacy (PR, TTP)	Boehringer lngelheim Pharma GmbH	Riechelmann 2008 [62]	
Bivatuzumab mertansine/BIWI-1	CD44v6	DM1	Cleavable disulfide	HNC	I	31/31	NS	Safety (CTC, DLT, MTD), PK profile, immunogenicity		Boehringer Ingelheim/ImmunoGen	Sauter 2007 [63]	

**CD44v6:** cluster of differentiation 44 variant domain 6; **CR:** complete response; **CTC:** NCI common toxicity criteria; **CTCAE:** common terminology criteria for adverse events; **DCR:** disease control rate; **DM1:** mertansine; **DOR:** duration of overall response; **DLT:** dose-limiting toxicity; **ECOG:** Eastern Cooperative Oncology Group; **EGFR:** epidermal growth factor receptor; **HER2:** human epidermal growth factor receptor 2; **HNC:** head and neck cancer; **MMAE:** monomethyl auristatin E; **MTD:** maximum tolerated dose; **NS:** not specified; **ORR:** objective response rate; **OS:** overall survival; **PD:** progressive disease; **PFS:** progression-free survival; **PR:** partial response; **SD:** stable disease; **TEAEs:** treatment-emergent adverse events; **TF:** tissue factor; **Trop-2:** trophoblast cell surface antigen 2; **TTP:** time to progression; **TTR:** time to response.

**Table 4:** Clinical Trials.

ADC	Target	Payload	Linker	Weblink	Trial Identifier/Study Phase	Sponsor	Status
ABBV-085	LRRC15	MMAE	Non-cleavable	https://clinicaltrials.gov/ct2/show/study/NCT02565758 (accessed on May 16th, 2021)	NCT02565758/I	AbbVie	Completed
A166	HER2	MMAF derivative	NS	https://clinicaltrials.gov/ct2/show/NCT03602079 (accessed on May 16th, 2021)	NCT03602079/I-II	Klus Pharma Inc.	Recruiting
CX-2029	CD71	MMAE	Valine-citrulline (VC) peptide	https://clinicaltrials.gov/ct2/show/NCT03543813 (accessed on May 16th, 2021)	NCT03543813/I-II	CytomX Therapeutics	Recruiting
CX-2009	CD71	MMAE	Valine-citrulline (VC) peptide	https://clinicaltrials.gov/ct2/show/NCT03149549 (accessed on May 16th, 2021)	NCT03149549/I-II	CytomX Therapeutics	Completed
SBT6050	HER2/TLR8	TLR8 agonist	NS	https://clinicaltrials.gov/ct2/show/NCT04460456 (accessed on May 16th, 2021)	NCT04460456/I	Silverback Therapeutics	Recruiting
ABBV-321	EGFR	PBD	Cathepsin-cleavable maleimidocaproyl-valine-alanine	https://clinicaltrials.gov/ct2/show/study/NCT03234712 (accessed on May 16th, 2021)	NCT03234712/I	AbbVie	Ongoing
MGC018	B7-H3 (CD276)	Synthetic duocarmycin analogs	Cleavable valine-citrulline-seco duocarmycin hydroxy-benzamide azaindole (vc-seco-DUBA)	https://clinicaltrials.gov/ct2/show/NCT03729596 (accessed on May 16th, 2021)	NCT03729596	MacroGenics	Recruiting
MRG003	EGFR	MMAE	NS	https://clinicaltrials.gov/ct2/show/NCT03729596 (accessed on May 16th, 2021)	NCT04868162	Shanghai Miracogen Inc.	Recruiting

**CD71:** cluster of differentiation 71; **HER2:** human epidermal growth factor receptor 2; **LRRC15:** membrane protein leucine-rich repeat containing 15; **MMAE:** monomethyl auristatin E; MMAF derivative: duostatin-5; **NS:** not specified; **TLR8:** Toll-like receptor 8; **vc-*seco*-DUBA**: valine-citrulline-*seco* duocarmycin hydroxybenzamide azaindole.

**Table 5:** Summarizes the design, approved indications and developer of ADCs approved by the FDA.

ADC	Target	Payload	Linker	Government Approval	Disease	Developer
Gemtuzumab ozogamicin	CD33	N-acetyl-γ calicheamicin 1,2-dimethyl hydrazine dichloride	4-(4-acetylphenoxy)butanoic acid (AcBut linker)	FDA	Relapsed acute myelogenous leukemia	Pfizer/Wyeth
Brentuximab vedotin	CD30	Monomethyl Auristatin E	Thiolreactive maleimidocaproyl spacer, the dipeptide valine–citrulline linker, and a self-immolative, p-amino-benzyloxycarbony (PABC) spacer	FDA	Hodgkin lymphoma and systemic anaplastic large-cell lymphoma	Seattle Genetics, Millennium/Takeda
Trastuzumab emtansine	HER2	Maytansinoid DM1	Non-reducible tioether linker: N-succinimidyl-4-(N-maleimidomethyl) cyclohexane-1-carboxylate linker (SMCC)	FDA	HER2-positive metastatic breast cancer following treatment with trastuzumab and a maytansinoid	Genentech, Roche
Inotuzumab ozogamicin	CD22	N-acetyl- γ Calicheamicin	Acid-labile(4-(4’-acetylphenoxy) butanoic acid) linker	FDA	Relapsed or refractory CD22-positive B-cell precursor acute lymphoblastic leukemia	Pfizer/Wyeth
Polatuzumab vedotin	CD79b	Monomethyl Auristatin E	Protease-cleavable peptide linker : maleimidocaproylvaline-citrulline-p-aminobenzoyloxycarbonyl linker (MC-VC-PABC)	FDA	Relapsed or refractory diffuse large B-cell lymphoma	Genentech, Roche
Enfortumab vedotin	Cell Surface Protein Nectin 4	Monomethyl Auristatin E	Protease-cleavable peptide linker : maleimidocaproylvaline-citrulline-p-aminobenzoyloxycarbonyl linker (MC-VC-PABC)	FDA	Adult patients with locally advanced or metastatic urothelial cancer who have received a PD-1 or PD-L1 inhibitor and a Pt-containing therapy	Astellas/Seattle Genetics
Trastuzumab deruxtecan	HER2	A topoisomerase I inhibitor payload, a derivative of the camptothecin analog exatecan (DXd)	A tetrapeptide linker, Gly-Phe-Leu-Gly (GFLG)	FDA	Adult patients with unresectable or metastatic HER2-positive breast cancer who have received two or more prior anti-HER2 based regimens	AstraZeneca/Daiichi Sankyo
Sacituzumab govitecan	TROP-2	SN-38 (active metabolite of irinotecan)	Hydrolyzable CL2A linker	FDA	Adult patients with metastatic triple-negative breast cancer who have received at least two prior therapies for patients with relapsed or refractory metastatic disease	Immunomedics
Belantamab mafodotin	TNFRSF17	Monomethyl Auristatin F	A non-cleavable maleimidocaproyl (MC) linker	FDA	Multiple myeloma patients whose disease has progressed despite prior treatment with an immunomodulatory agent, proteasome inhibitor and anti-CD38 antibody	GlaxoSmithKline
Loncastuximab tesirine	CD19	SG3199/ Pyrrolobenzodiazepine (PBD) dimer SCX	A cleavable (valine-alanine dipeptide as cathepsin B cleavage site) maleimide type linker containing a PEG spacer	Japan	Relapsed or refractory large B-cell lymphoma (including diffuse large B-cell lymphoma not otherwise specified, arising from low-grade lymphoma, and high-grade B-cell lymphoma) after two or more lines of systemic therapy	ADC Therapeutics
